# Challenging Management of a Rare Complex Cerebral Arteriovenous Malformation in the Corpus Callosum and Post-Central Gyrus: A Case Study of a 41-Year-Old Female

**DOI:** 10.3390/jcm13247494

**Published:** 2024-12-10

**Authors:** Corneliu Toader, Felix Mircea Brehar, Mugurel Petrinel Radoi, Razvan Adrian Covache-Busuioc, Matei Serban, Alexandru Vladimir Ciurea, Nicolaie Dobrin

**Affiliations:** 1 Department of Neurosurgery, “Carol Davila” University of Medicine and Pharmacy, 050474 Bucharest, Romania; corneliu.toader@umfcd.ro (C.T.); felix.brehar@umfcd.ro (F.M.B.); petrinel.radoi@umfcd.ro (M.P.R.); razvan-adrian.covache-busuioc0720@stud.umfcd.ro (R.A.C.-B.); prof.avciurea@gmail.com (A.V.C.); 2 Department of Vascular Neurosurgery, National Institute of Neurology and Neurovascular Diseases, 077160 Bucharest, Romania; 3 Department of Neurosurgery, Clinical Emergency Hospital “Bagdasar-Arseni”, 041915 Bucharest, Romania; 4 Department of Neurosurgery, Sanador Clinical Hospital, 010991 Bucharest, Romania; 5 “Nicolae Oblu” Clinical Hospital, 700309 Iasi, Romania; dobrin_nicolaie@yahoo.com

**Keywords:** cerebral arteriovenous malformations (AVMs), corpus callosum, post-central gyrus, hemorrhagic stroke, microsurgical resection, deep venous drainage, anterior cerebral artery (ACA), intraoperative monitoring, Spetzler-Martin grade

## Abstract

**Background/Objectives:** Cerebral arteriovenous malformations (AVMs) are rare but complex vascular anomalies, particularly challenging when located in eloquent regions such as the corpus callosum and post-central gyrus. This report aims to highlight the management and outcomes of a 41-year-old female patient with a hemorrhagic AVM in these critical areas, emphasizing the importance of early surgical intervention and advanced imaging techniques. **Methods:** The patient presented with a right-sided tonic–clonic seizure and expressive aphasia, prompting imaging that revealed a complex AVM with deep venous drainage and arterial supply from the anterior cerebral artery. A multidisciplinary team performed microsurgical resection via a left parasagittal fronto-parietal craniotomy. The surgical approach prioritized hematoma evacuation followed by a stepwise dissection of the AVM nidus under intraoperative monitoring. **Results:** Complete resection of the AVM was confirmed through postoperative angiographic and CT imaging. The patient showed stable recovery over 15 months, with no recurrence or new neurological deficits. This case demonstrates the critical role of advanced imaging, intraoperative strategies, and a multidisciplinary approach in achieving successful outcomes. **Conclusions:** Microsurgical resection remains the gold standard for AVMs in eloquent and deep-seated brain regions. Early diagnosis and tailored surgical interventions are crucial for managing these high-risk cases. This case underscores the importance of integrating advanced imaging, strategic surgical planning, and intraoperative monitoring to minimize complications and optimize long-term recovery.

## 1. Introduction

Cerebral arteriovenous malformations (AVMs) are rare vascular anomalies characterized by direct connections between arteries and veins, bypassing the normal capillary system. This malformation results in abnormal blood flow dynamics, leading to potential rupture and hemorrhage [1]. AVMs make up around 10–15% of intracranial vascular malformations and pose significant risks, particularly in younger individuals. These lesions often remain asymptomatic until complications, such as hemorrhage or seizures, arise, making early detection and management crucial for patient outcomes [2].

Cerebral AVMs located in the corpus callosum are often classified as eloquent due to their proximity to structures critical for interhemispheric communication and cognitive functions [3]. The eloquence of these AVMs poses significant challenges during surgical planning and management, requiring a balance between achieving complete resection and preserving neurological function [4].

AVMs located within the corpus callosum (CC-AVMs) are notably rare, accounting for approximately 8–9% of all cerebral AVMs [5]. The corpus callosum, being a central structure responsible for interhemispheric communication, makes these AVMs particularly unique. The arterial supply for CC-AVMs frequently arises from branches of the anterior cerebral artery (ACA), especially the pericallosal artery [6,7]. Their venous drainage commonly involves deep venous systems, such as the internal cerebral veins or the vein of Galen [8]. The clinical presentation of CC-AVMs varies based on the size and location of the malformation, with hemorrhage being the most frequent initial presentation, occurring in up to 80% of cases.

Hemorrhages associated with CC-AVMs tend to be more severe, often leading to intraventricular hemorrhages and hemohydrocephalus due to the deep venous drainage into critical structures, such as the ventricles [9]. In addition to hemorrhages, CC-AVMs may present with seizures in up to 20–29% of cases. These seizures can be focal or generalized, and they are often linked to increased venous pressure, ischemia from steal phenomena, or previous intracerebral hemorrhages (ICHs) [6]. The diagnosis of CC-AVMs relies heavily on imaging techniques, with magnetic resonance imaging (MRI) being the gold standard for evaluating non-hemorrhagic presentations. In cases where hemorrhage is suspected, computed tomography (CT) is typically the first imaging modality used, followed by digital subtraction angiography (DSA) to assess the AVM’s angioarchitecture and venous drainage [10].

Treatment modalities for CC-AVMs are multifaceted and depend on factors such as the Spetzler-Martin grade, patient age, and the AVM’s proximity to eloquent brain regions [11]. Treatment options include microsurgical resection, endovascular embolization, and stereotactic radiosurgery, each with its own risk–benefit profile. Given the deep location and potential involvement of critical brain structures, complete surgical resection may be challenging, often requiring multimodal approaches to reduce the risk of neurological deficits [10].

## 2. Case Presentation

The 41-year-old female patient was admitted to our clinic following a right-sided tonic–clonic seizure with secondary generalization. This event resulted in a traumatic brain injury caused by a fall from the same height, which was accompanied by expressive aphasia.

The native MRI examination reveals a recent left fronto-parietal intraparenchymal hematoma (marked by hypointense T2 signal with hyperintense inclusions on 3D TOF-T1 sequences) accompanied by mild reactive perilesional edema. This hematoma exerts a slight mass effect on the atrium of the left lateral ventricle, without causing a midline shift. The examination also identifies draining veins located medially to the hematoma, draining into the superior sagittal sinus, as well as feeding arteries originating from the ACA.

**Figure 1:**
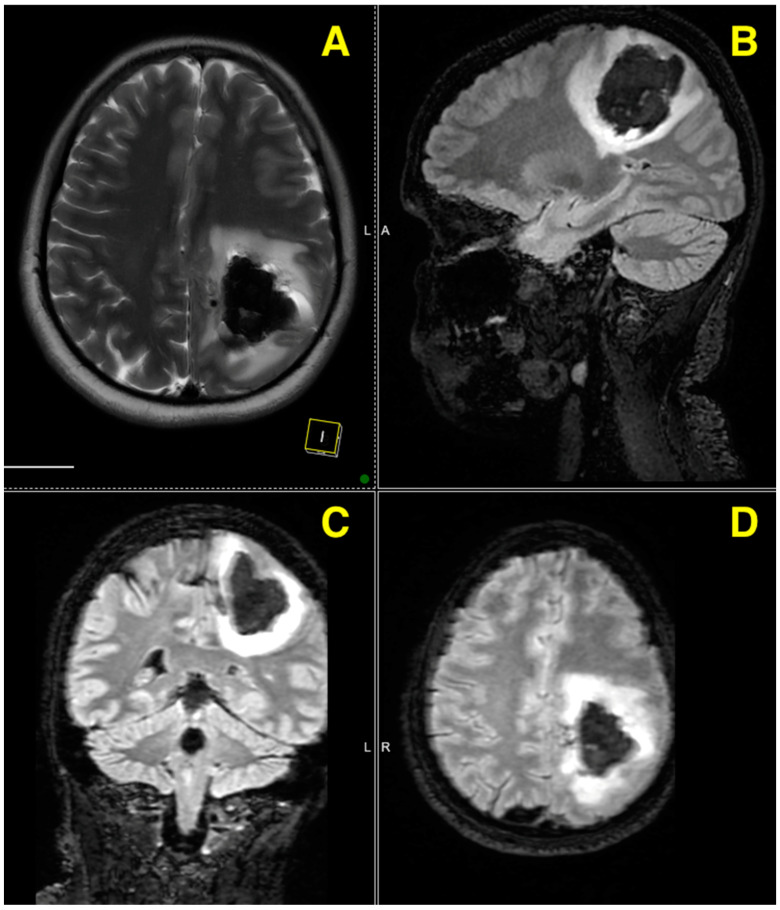
Four different MRI images from a preoperative scan provide a detailed view of a left fronto-parietal lesion. (**A**) is an axial T2-weighted view, which presents a horizontal cross-section of the brain, offering a detailed look at the lesion’s involvement in the left fronto-parietal region. (**B**) is a sagittal T2-weighted view, providing a vertical cross-section of the brain from the side, which highlights the vertical extent of the lesion and its relationship with the ventricular system. (**C**) is a coronal T2-weighted view, showing a frontal cross-section of the brain, demonstrating the lesion’s depth and impact on the surrounding cortical and subcortical structures. (**D**) is an axial diffusion-weighted imaging (DWI) view, presenting a horizontal cross-section that emphasizes the diffusion characteristics of the tissue around the lesion, revealing restricted diffusion related to the hemorrhagic event.

The MRI images reveal a recent left fronto-parietal intraparenchymal hematoma, which is visible on the T2-weighted sequence as a hypointense region, with hyperintense inclusions seen in 3D TOF-T1 sequences (Figure 1). The lesion is accompanied by mild reactive perilesional edema, exerting a slight mass effect on the adjacent structures, specifically the atrium of the left lateral ventricle, though without causing a significant midline shift. Additionally, draining veins are identified medially to the hematoma, draining into the superior sagittal sinus, while the feeding arteries originate from the ACA.

The axial, sagittal, and coronal views further demonstrate the lesion’s extension and its mass effect on nearby brain structures. The increased signal intensity on the diffusion-weighted imaging (DWI) is suggestive of restricted diffusion, likely due to cytotoxic edema or residual hemorrhagic components, correlating well with the known hemorrhagic nature of this lesion. These findings unambiguously correlate with a recent hemorrhagic event, definitively caused by a vascular malformation.

The images conclusively show the involvement of both cortical and subcortical regions, as well as the adjacent ventricular structures. The lesion’s location and characteristics, coupled with the imaging evidence, strongly indicate a vascular etiology.

**Figure 2:**
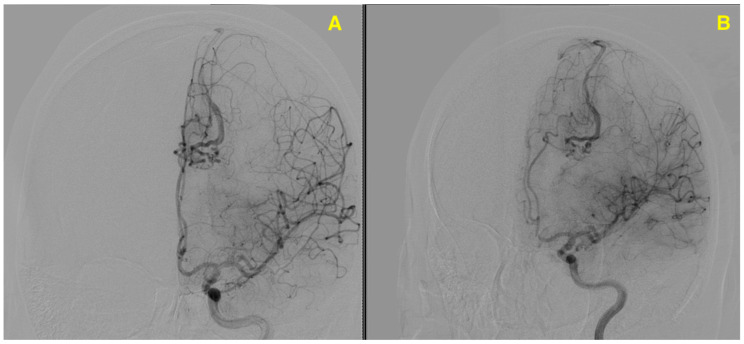
Provides two lateral angiographic views that highlight the vascular structure of the left fronto-parietal AVM. (**A**) Arterial phase highlights the intricate network of abnormal vessels, with direct arteriovenous shunting. Feeding arteries arise predominantly from branches of the anterior cerebral artery (ACA), with abnormal venous drainage into dilated structures converging on the superior sagittal sinus. (**B**) Late arterial/early venous phase shows reduced vascular filling, indicating flow dynamics associated with the AVM. These findings correlate with the hemorrhagic event observed on preoperative MRI, providing critical insights for surgical planning.

The patient underwent an angiographic scan to further evaluate the vascular structures associated with the hemorrhagic lesion observed on the preoperative MRI. The angiography revealed a complex AVM located in the left fronto-parietal region, with feeding arteries originating from branches of the ACA. This scan highlighted the direct arteriovenous shunting, with abnormal drainage into dilated venous structures, consistent with the findings on the MRI. The abnormal vascular network, clearly visible in the angiographic images, correlates with the hemorrhagic event seen in the MRI (Figure 2). The second angiographic image suggests reduced vascular filling, indicating partial resolution or intervention-related changes, consistent with post-treatment status.

**Figure 3:**
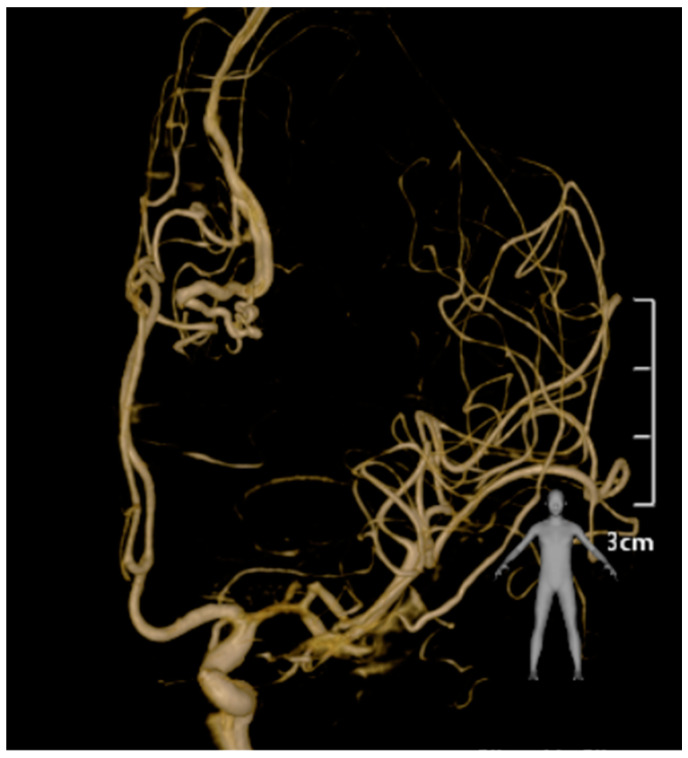
This figure presents a preoperative lateral angiographic image that illustrates the arterial phase of a left fronto-parietal AVM. The image reveals an intricate tangle of abnormal vessels typical of an AVM, with feeding arteries originating from branches of the ACA. The absence of a normal capillary bed is evident, and there is prominent arteriovenous shunting. The draining veins are directed toward the superior sagittal sinus, corresponding to the hemorrhagic lesion observed on the preoperative MRI. This angiographic view is crucial in characterizing the AVM and its hemodynamic properties prior to surgical intervention.

DSA provided a comprehensive visualization of the AVM’s intricate angioarchitecture, delineating both its arterial supply and venous drainage patterns. The primary arterial feeders originated from the pericallosal branch of the ACA, which formed the dominant inflow to the nidus. Additionally, minor feeders were identified from cortical branches of the middle cerebral artery (MCA), contributing supplementary flow to the malformation. The venous drainage was characterized as exclusively deep, with enlarged and tortuous draining veins converging medially into the superior sagittal sinus. The flow dynamics within the AVM were notably high, reflecting the hemodynamic burden imposed by the direct arteriovenous shunting. This hemodynamic profile posed significant challenges, particularly the risk of intraoperative venous congestion and hemorrhagic complications. These findings underscored the importance of preoperative planning and intraoperative strategies tailored to manage high-flow AVMs while minimizing the risk of neurological compromise. The detailed vascular mapping provided by DSA was critical in guiding surgical dissection and ensuring a controlled resection of the nidus (Figure 3).

The AVM was classified as Spetzler-Martin (SM) Grade III, reflecting its intermediate complexity due to its small size, location in eloquent regions such as the corpus callosum and post-central gyrus, and deep venous drainage into the superior sagittal sinus. This grading emphasized the critical need for meticulous surgical planning to achieve complete resection while preserving essential neurological functions. The SM Grade III designation guided the approach to this challenging case, highlighting the delicate balance between effective treatment and minimizing the risk of complications.

The surgical procedure for this case was an extraordinary example of a meticulously tailored approach designed to address the unique challenges posed by a Spetzler-Martin Grade III AVM located in the corpus callosum and post-central gyrus. These regions are critically important for motor function and interhemispheric communication, making the surgery inherently high-risk and requiring innovative strategies to achieve success. The operation began with a left parasagittal fronto-parietal craniotomy, strategically oriented to provide direct access to the deep-seated lesion while preserving the superior sagittal sinus and minimizing disruption to adjacent eloquent areas. This craniotomy design allowed for a controlled and precise entry, ensuring optimal exposure to the AVM’s vascular network without compromising critical cortical or subcortical structures.

A major highlight of the procedure, showcasing its novelty, was the prioritization of hematoma evacuation as the first step. Unlike traditional AVM surgeries that often focus directly on the nidus, this approach provided significant advantages. The early removal of the hemorrhagic clot decompresses brain tissue, alleviating intracranial pressure and reducing the risk of secondary ischemia in adjacent eloquent regions. Moreover, this step enhanced the visibility of the AVM’s complex angioarchitecture, enabling safer and more precise dissection of its feeders and venous drainage pathways. This strategic sequence exemplifies a novel integration of hematoma management into AVM surgery, improving safety and procedural efficiency.

The AVM nidus, located on the medial surface of the post-central gyrus, presented significant challenges due to its proximity to critical motor pathways. Under the guidance of a high-powered operating microscope, the nidus was carefully dissected circumferentially. The surgical team employed an innovative stepwise vascular management strategy, sequentially coagulating and dividing feeder vessels primarily originating from the pericallosal artery. This gradual reduction in inflow to the AVM ensured hemodynamic stability and minimized the risk of intraoperative hemorrhage, a common complication in high-flow AVM resections. The draining vein, a large, engorged, and reddish structure, was preserved until the nidus was entirely excised to prevent venous congestion. Its subsequent reduction in size and color changes to bluish confirmed the cessation of abnormal blood flow and restoration of normal venous dynamics.

What sets this case apart is the innovative combination of techniques and strategic decision-making tailored to the AVM’s unique characteristics. The prioritization of hematoma evacuation provided a novel foundation for the entire procedure, transforming the surgical field and significantly reducing risks. The stepwise feeder disconnection method exemplifies an advanced approach to managing high-flow hemodynamics, while the strategic preservation of the draining vein highlighted a deep understanding of the lesion’s vascular dynamics. Together, these elements underscore a paradigm shift in how complex AVMs, particularly those in eloquent regions, can be approached.

**Figure 4:**
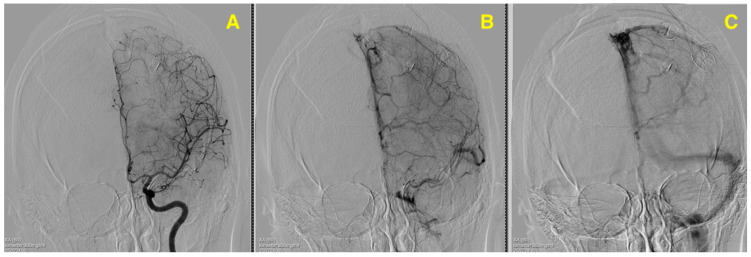
(**A**) highlights the arterial phase, showing the absence of abnormal arterial feeders from the anterior cerebral artery, which previously supplied the AVM. (**B**) displays the venous phase, where the normalization of the venous drainage into the superior sagittal sinus is evident, with no abnormal venous structures visible. (**C**) demonstrates the delayed phase, confirming that no residual AVM or abnormal vascular connections remain postoperatively. This angiographic follow-up provides definitive evidence of successful AVM resection, with no complications or residual vascular abnormalities noted.

The outcome was a resounding success. The AVM nidus, measuring approximately 2.5 cm in diameter, was completely excised without residual malformation, as confirmed by postoperative angiography. The patient experienced no new neurological deficits, and her recovery trajectory remained stable over 15 months. This case exemplifies the integration of innovative surgical techniques with a deep understanding of vascular anatomy and dynamics, providing a valuable model for future AVM management in similarly challenging cases (Figure 4).

**Figure 5:**
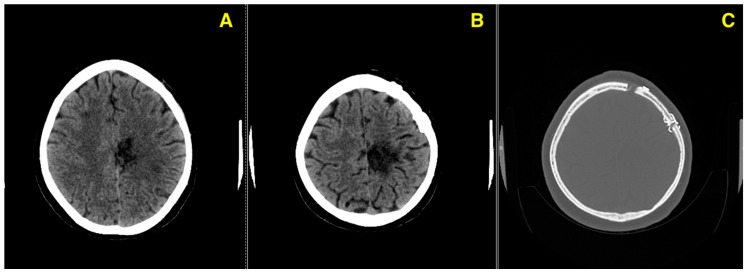
This figure presents three axial CT images taken at the 3-month follow-up, evaluating the patient’s postoperative condition following the excision of a left fronto-parietal AVM. (**A**): Axial CT image demonstrating normal postoperative parenchymal appearance in the left fronto-parietal region, with no evidence of residual AVM, hemorrhage, or ischemic changes. (**B**): Axial CT image focusing on the soft tissue and adjacent brain structures, confirming the absence of edema or any abnormal enhancement around the surgical site. (**C**): Bone window CT image evaluating the integrity of the bone flap, showing proper placement and no signs of hardware failure, infection, or other complications.

The 3-month follow-up CT (Figure 5) confirms a stable postoperative course, with no evidence of complications such as hemorrhage, ischemia, or issues with the bone flap, highlighting the patient’s continued recovery.

**Figure 6:**
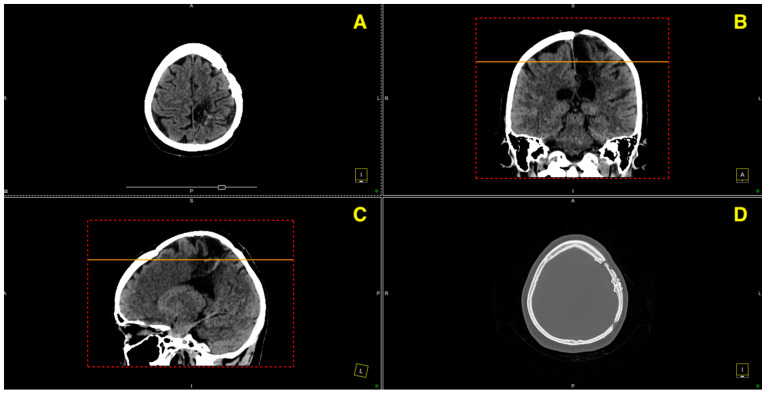
This figure presents a series of axial, coronal, and sagittal CT images, alongside a bone window, from the 12-month follow-up evaluation post-excision of a left fronto-parietal AVM. (**A**) Axial view showing the absence of residual AVM or hemorrhagic changes in the left fronto-parietal region, confirming the stability of the surgical site. (**B**) Coronal view illustrating the preserved brain structures with no signs of edema, recurrence, or new vascular anomalies. (**C**) Sagittal view highlighting the absence of mass effect or midline shift, with normal postoperative anatomy. (**D**) Bone window axial view verifying the integrity of the craniotomy site, with no complications such as bone resorption or infection.

The 12-month follow-up CT (Figure 6) demonstrates a stable postoperative course with no evidence of recurrence, residual AVM, or post-surgical complications. The imaging findings indicate a successful long-term outcome, with continued clinical improvement and no new neurological deficits or pathological developments.

**Figure 7:**
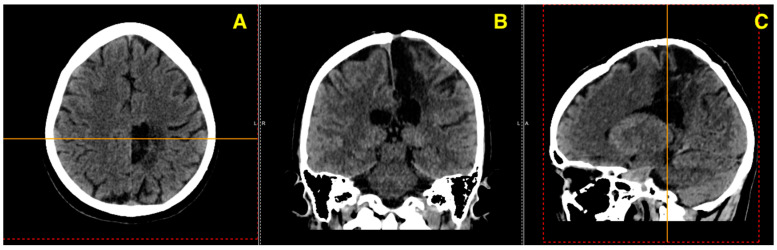
These images together confirm a favorable outcome, with no significant complications observed at the 15-month mark, supporting a good recovery trajectory for the patient. (**A**) Axial view reveals a stable postoperative site in the left fronto-parietal region, with no signs of residual AVM or recurrent hemorrhage. (**B**) Coronal view illustrates symmetrical ventricular structures and preserved parenchyma, with no evidence of new lesions or complications. (**C**) Sagittal view confirms the absence of pathological developments, with normal postoperative anatomy and no mass effect or midline shift.

The patient’s 15-month follow-up CT scan (Figure 7) provides an essential evaluation of the postoperative progress following the excision of a left fronto-parietal AVM. This imaging study demonstrates clear improvements and stable recovery, showcasing the long-term success of the surgical intervention.

## 3. Discussion

Cerebral AVMs are complex vascular malformations that pose significant clinical and management challenges, particularly when located in eloquent or deep brain regions such as the corpus callosum and post-central gyrus [12]. AVMs in these regions are rare, comprising only about 8–9% of all cerebral AVMs, and they often present with significant morbidity due to the high risk of rupture and hemorrhage [13]. This case report details the successful management of a large corpus callosum AVM in a 41-year-old female who presented with a hemorrhagic event and highlights the intricacies of the surgical intervention and follow-up in alignment with the current literature. The majority of AVMs, particularly those in deep or eloquent brain regions, present with hemorrhage, which is the leading cause of morbidity and mortality associated with these lesions [14]. This is consistent with the presentation in this case, where the patient experienced a left fronto-parietal hemorrhage associated with the AVM [15]. Studies indicate that up to 80% of corpus callosum AVMs present with hemorrhage, often resulting in intraventricular hemorrhage or intracerebral hematoma due to the deep venous drainage involved [16].

The deep venous drainage into the superior sagittal sinus and the involvement of the pericallosal artery further contributed to the complexity of this case. In cases of ruptured AVMs, microsurgical resection remains the gold standard, especially for large, deep-seated lesions where other modalities, such as endovascular embolization or radiosurgery, may not achieve complete obliteration of the AVM [17]. The decision to proceed with a left parasagittal fronto-parietal craniotomy, in this case, was driven by the need to achieve complete resection of the AVM to prevent future hemorrhages, as partial treatments have been associated with higher risks of rebleeding and incomplete resolution. The literature supports the need for early surgical intervention in such cases, particularly when the AVM has already caused significant hemorrhagic complications [18]. During surgery, the primary goal was to achieve a complete excision of the AVM while minimizing damage to adjacent eloquent areas, such as the motor cortex, and preserving normal brain tissue. Microsurgical resection remains the most definitive treatment modality for AVMs of this nature, especially for those involving the corpus callosum, where preserving cognitive and motor function is of utmost importance.

Recent studies emphasize the importance of intraoperative monitoring and the use of microsurgical techniques to ensure that eloquent areas are not damaged during AVM resection. The successful excision of a 2.5 cm nidus, in this case, without any intraoperative complications, supports the effectiveness of these techniques in achieving complete AVM resection while minimizing the risks of new neurological deficits. Recent studies indicate that complete resection of AVMs, confirmed by early postoperative imaging, significantly reduces the risk of recurrence and rebleeding, particularly in high-risk AVMs like those located in deep brain structures [19]. The stable follow-up imaging over a period of 15 months in this case aligns with these findings, highlighting the importance of meticulous surgical technique and thorough postoperative monitoring in ensuring long-term success. While this case highlights the success of microsurgical resection, it is important to recognize that multimodal approaches to AVM treatment, including endovascular embolization and radiosurgery, have become more common in recent years. These modalities are particularly useful in cases where surgical resection carries high risks, or where AVMs are located in areas not easily accessible by surgery. However, in deep-seated AVMs like the one presented here, microsurgical resection remains the definitive treatment to prevent future hemorrhagic events, as embolization alone is often insufficient for achieving complete obliteration of the AVM.

The eloquence of CC-AVMs stems from their location within the corpus callosum, a critical structure mediating interhemispheric communication and cognitive integration. Damage to this region can result in profound neurocognitive and motor deficits, emphasizing the need for meticulous surgical planning [20]. The unique challenges of CC-AVM resection arise from their deep-seated position and adjacency to other eloquent brain regions, necessitating the use of advanced intraoperative modalities such as neuronavigation, cortical and subcortical mapping, and real-time electrophysiological monitoring [21]. These tools ensure maximal preservation of neural function while enabling safe nidus resection. The functional eloquence of CC-AVMs mandates a tailored surgical approach that minimizes collateral damage, underscoring the importance of individualized strategies that integrate preoperative imaging, angioarchitectural analysis, and intraoperative precision [22].

Recent advances in imaging and surgical techniques have further improved the outcomes of AVM resections. The use of intraoperative angiography, neuronavigation, and real-time monitoring of cortical function during surgery has enhanced the safety and effectiveness of AVM resections, particularly in eloquent or deep brain regions. Understanding the various factors influencing treatment outcomes, such as AVM location, size, and the chosen therapeutic approach, is critical for improving patient care (Table 1).

## 4. Conclusions

This case report highlights the effective treatment of a complex AVM in the corpus callosum and post-central gyrus of a 41-year-old female. The patient presented with a significant hemorrhagic event, reflecting the high risks associated with AVMs in deep brain structures. Successful management was achieved through early diagnosis and a tailored surgical approach, leading to complete resection and favorable long-term outcomes, as confirmed by follow-up imaging. Microsurgical resection was crucial in this case, proving to be the most reliable treatment for ensuring complete removal and reducing the risk of recurrence. The use of advanced intraoperative techniques and detailed postoperative monitoring contributed to the patient’s stable recovery, with no new neurological deficits. This case reinforces the importance of a multidisciplinary and strategic approach to treating high-risk AVMs, particularly in critical brain areas, and highlights the advancements in surgical and imaging technology that enhance patient outcomes.

## Figures and Tables

**Table 1:** This table provides a comparative summary of various studies on arteriovenous malformations (AVMs), focusing on patient demographics, AVM characteristics, treatment options, and complications. The studies, spanning from 2015 to 2024, involve patient populations ranging from 109 to 300, with a relatively consistent male-to-female ratio of approximately 1.1 to 1.3. AVM locations include the anterior cerebral, middle cerebral, pericallosal, and basilar arteries, with diameters ranging from 1 to 5 cm.

Authors and Year	Number of Patients	Sex Ratio (M:F)	AVM Artery	AVM Diameter	Treatment Option	Complications
Liyis et al., 2024 [13]	112	1.2:1	Pericallosal, posterior cerebral	1.5–5 cm	Microsurgery, embolization	5% hemorrhage, 4% new neurological deficits
Brosnan et al., 2022 [23]	250	1.1:1	Anterior cerebral, MCA	2–5 cm	Multimodal: embolization, microsurgery	18.8% death or dependency, 13.2% neurological deficits
Park et al., 2022 [24]	National Sample	1.2:1	MCA, posterior cerebral	2–4 cm	Microsurgery, embolization, radiosurgery	3.9% annual stroke risk for medical management vs. 12.5% for interventional treatment
Mohr et al., 2020 [25]	109	1.1:1	MCA, basilar artery	2–5 cm	Conservative treatment, intervention (microsurgery, embolization)	30.7% stroke/death in the intervention group vs. 10.1% in the medical group
Lawton et al., 2015 [26]	300	1.3:1	MCA, anterior cerebral, posterior cerebral	1.5–4.5 cm	Microsurgery, preoperative embolization	8% recurrence, 5% postoperative deficits

## Data Availability

The data presented in this study are available on request from the corresponding author.
